# Influences of Sr^2+^ Doping on Microstructure, Giant Dielectric Behavior, and Non-Ohmic Properties of CaCu_3_Ti_4_O_12_/CaTiO_3_ Ceramic Composites

**DOI:** 10.3390/molecules26071994

**Published:** 2021-04-01

**Authors:** Jutapol Jumpatam, Bundit Putasaeng, Narong Chanlek, Prasit Thongbai

**Affiliations:** 1 Department of Fundamental Science, Faculty of Science and Technology, Surindra Rajabhat University, Surin 32000, Thailand; Jutapoljum@outlook.com; 2 National Metal and Materials Technology Center (MTEC), Thailand Science Park, Pathumthani 12120, Thailand; bunditp@mtec.or.th; 3 Synchrotron Light Research Institute (Public Organization), 111 University Avenue, Muang District, Nakhon Ratchasima 30000, Thailand; Narong@slri.or.th; 4 Department of Physics, Faculty of Science, Khon Kaen University, Khon Kaen 40002, Thailand; 5 Institute of Nanomaterials Research and Innovation for Energy (IN-RIE), Khon Kaen University, Khon Kaen 40002, Thailand

**Keywords:** ceramic composite, CaCu_3_Ti_4_O_12_/CaTiO_3_, dielectric permittivity, non-Ohmic properties, loss tangent

## Abstract

The microstructure, dielectric response, and nonlinear current-voltage properties of Sr^2+^-doped CaCu_3_Ti_4_O_12_/CaTiO_3_ (CCTO/CTO) ceramic composites, which were prepared by a solid-state reaction method using a single step from the starting nominal composition of CCTO/CTO/xSrO, were investigated. The CCTO and CTO phases were detected in the X-ray diffraction patterns. The lattice parameter increased with increasing Sr^2+^ doping concentration. The phase compositions of CCTO and CTO were confirmed by energy-dispersive X-ray spectroscopy with elemental mapping in the sintered ceramics. It can be confirmed that most of the Sr^2+^ ions substituted into the CTO phase, while some minor portion substituted into the CCTO phase. Furthermore, small segregation of Cu-rich was observed along the grain boundaries. The dielectric permittivity of the CCTO/CTO composite slightly decreased by doping with Sr^2+^, while the loss tangent was greatly reduced. Furthermore, the dielectric properties in a high-temperature range of the Sr^2+^-doped CCTO/CTO ceramic composites can be improved. Interestingly, the nonlinear electrical properties of the Sr^2+^-doped CCTO/CTO ceramic composites were significantly enhanced. The improved dielectric and nonlinear electrical properties of the Sr^2+^-doped CCTO/CTO ceramic composites were explained by the enhancement of the electrical properties of the internal interfaces.

## 1. Introduction

Over the last decades, giant dielectric materials with high dielectric permittivity (ε′ > 10^3^) have been continuously investigated to develop ceramic capacitors and high energy density storage applications. This is due to a growing demand for miniaturization in microelectronics with the emergence of portable electronic device industry applications (e.g., smartphones and tablets), including applications in the automotive and aerospace industries. CaCu_3_Ti_4_O_12_ (CCTO) has been extensively studied in the field of high-permittivity dielectric materials. CCTO is one of the most of dielectric oxides in the ACu_3_Ti_4_O_12_ family, which can exhibit very high ε′ over a wide range from 100 K to 400 K [1,2,3,4,5,6,7,8,9,10,11]. Unfortunately, the dielectric loss tangent of ACu_3_Ti_4_O_12_ ceramics (tanδ > 0.1) is usually higher than the standard tanδ value.

Besides the giant dielectric properties, CCTO and related ACu_3_Ti_4_O_12_ ceramics exhibited attractive nonlinear current density-electric field (J-E) properties or non-Ohmic properties [3,4,9,12,13]. Thus, CCTO and related ACu_3_Ti_4_O_12_ ceramics can be used in varistor devices when the non-Ohmic parameters can be enhanced.

Although the exact origin of giant ε′ for the CCTO is still unclear, it has been widely accepted that the extrinsic effect of internal interfaces is the primary cause of the giant dielectric response and non-Ohmic properties in polycrystalline CCTO-based ceramics [14,15,16]. For the CCTO-based ceramics, the observed heterogeneous electrical microstructure, consisting of insulating grain boundaries (GBs) sandwiched by semiconducting grains, are supported the internal (GB) barrier layer capacitor (IBLC) effect [1,5,7,12]. However, the intrinsic effect of the grains cannot be ignored or illogically excluded [17]. The dielectric response and related nonlinear electrical behavior result from the Schottky potential barrier at the interface between adjacent semiconducting grains [18,19,20,21].

Various approaches for improving the dielectric properties, i.e., reducing the low-frequency tanδ value and optimizing the non-Ohmic characteristics of CCTO ceramics, have been widely proposed, such as doping and co-doping with various metal ions into Ca^2+^, Cu^2+^, and Ti^4+^ sites in the CCTO structure [6,7,8,9,22,23]; fabrication a dense, fine grain-sized microstructure using a chemical synthesis [24,25]; or fabrication of the composite ceramics [4,18,20,21,26]. The CCTO-based composites, incorporating a highly insulating phase such as CaTiO_3_ (CTO), have been widely fabricated to optimize the dielectric and nonlinear electrical properties [18,19,26]. Using a one-step process from the ceramic powder with a nominal chemical formula of CCTO/CTO, the ceramic composite consisting of 66.7 mol% of CCTO and 33.3 mol% of CTO can be obtained. The CCTO/CTO composites show a decreased tanδ value (~0.02), with ε′ about of 1800 at 1 kHz and room temperature (RT) [19,21,27,28]. Moreover, the breakdown electric field (E_b_) and nonlinear coefficient (α) of the CCTO/CTO composite ceramics are also enhanced compared to the single-phase CCTO ceramics. It was reported that low tanδ and giant ε′ in CCTO/CTO composite could be achieved by doping Mg^2+^ [29], Zn^2+^ [18], and Sn^4+^ [20,26]. Substitution of Sr^2+^ into the CCTO ceramics has been widely studied due to the impressive dielectric results [2,8,9]. A significantly decreased tanδ with retaining a high ε′ was obtained in the Sr^2+^-doped CCTO ceramics [8]. For capacitor applications, tanδ of a dielectric material should be reduced as low as possible to prevent the dissipation of energy, while *E*_b_ must be increased as high as possible for application in a high voltage level. Sr^2+^ doping ions can improve the electrical properties of the internal interface (i.e., GBs) of CCTO ceramics, giving rise to the enhanced dielectric and nonlinear electrical properties [8,23,30,31]. Thus, using this concept, the objective of this work is to reduce tanδ of the CCTO/CTO composites with enhancing the nonlinear electrical properties by doping with Sr^2+^ ions. To the best of our knowledge, the influences of Sr^2+^ doping on microstructure, dielectric response, and non-Ohmic properties in CCTO/CTO composite systems have never been reported.

In this work, the Sr^2+^-doped CCTO/CTO ceramic composites are prepared using a nominal chemical formula of CCTO/CTO/xSrO. The phase formation and compositions are systematically investigated. The electrical and dielectric properties are studied and discussed in detail. The expected results of Sr^2+^-doped CCTO/CTO composite systems may satisfy a promise for practical ceramic capacitors and high energy density storage applications.

## 2. Results and Discussion

**Figure 1:**
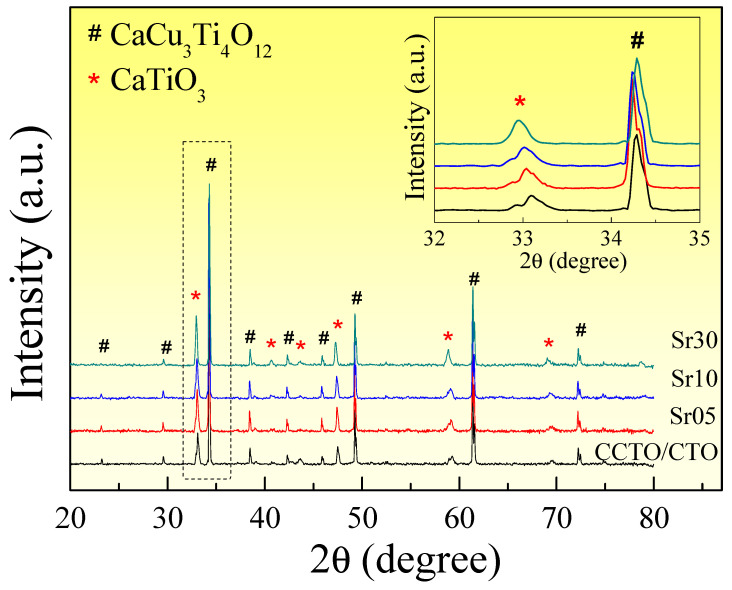
X-ray diffraction (XRD) patterns of all composite samples; inset shows shifting XRD peak ~33.0° for the CaTiO_3_ (CTO) phase.

The phase compositions of Sr^2+^-doped CCTO/CTO ceramic composites sintered at 1100 °C for 5 h were investigated using the X-ray diffraction (XRD) technique, as illustrated in Figure 1. Two primary phases of CCTO (JCPDS 75-2188) and CTO (JCPDS 82-0231) were detected in the XRD patterns of all the composites. No possible impurity phase, e.g., CuO or SrTiO_3_, was observed. This observation is similar to those reported in the literature for the CCTO/CTO composites [20,21,26,28,32], which comprised of ~66.7 mol% CCTO and ~33.3 mol% CTO [18,28,29]. The nominal composition of CCTO/CTO can be written as Ca(Cu_2_Ca)Ti_4_O_12_, which is similar to that of CCTO. However, the creation of the CCTO/CTO composite occurred due to a very larger ionic radius of Ca^2+^ (>1.00 Å) compared to that of the Cu^2+^ (0.57 Å) [33]. Excess Ca^2+^ ions could not occupy the Cu^2+^ sites. The calculated lattice parameter (*a*) values of the CCTO phase in all the composites are in the range of 7.391–7.392 Å. As illustrated in the inset of Figure 1, the main peak of the CTO phase in the Sr^2+^-doped CCTO/CTO ceramic composites shifted to a low 2θ angle with increasing Sr^2+^ content, indicating the increase in cell parameters of the CTO phase. This result was due to the larger ionic radius of Sr^2+^ (*r*_12_ = 1.44 Å) compared to Ca^2+^ (*r*_12_ = 1.34 Å) ions [33]. Therefore, Sr^2+^ doping ions were likely to prefer substitution into the CTO structure.

**Figure 2:**
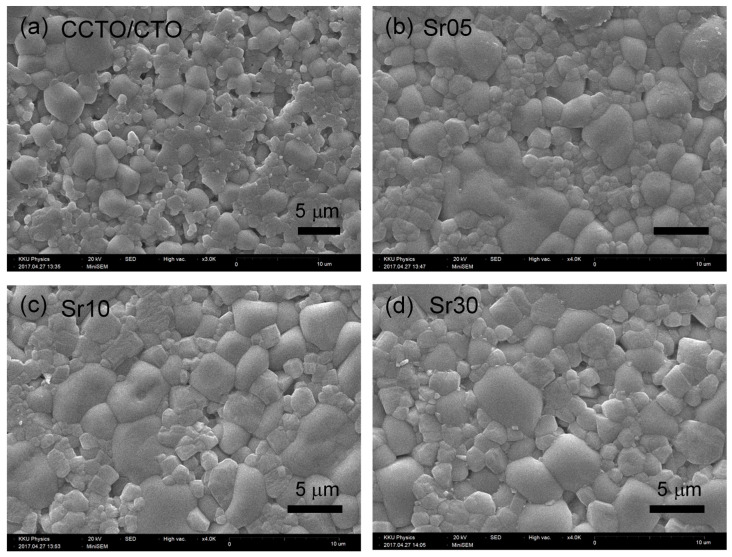
Scanning electron microscopy (SEM) images of Sr^2+^-doped CaCu_3_Ti_4_O_12_ (CCTO)/CTO composites: (**a**) CCTO/CTO, (**b**) Sr05, (**c**) Sr10, and (**d**) Sr30 composite samples.

Figure 2 shows the surface morphologies of the CCTO/CTO and Sr^2+^-doped CCTO/CTO ceramic composites. Two sets of grain shapes were observed, i.e., large grains with a smooth surface and small grains with a rough surface. According to the previous reports [21,28], the smooth and rough grains are suggested to be the CCTO and CTO phases, respectively. It was observed that the grain size of the smooth grains of the CCTO/CTO composites tended to become enlarged by doping with Sr^2+^ ions, corresponding to that observed in the Sr^2+^-doped CCTO ceramics [8].

**Figure 3:**
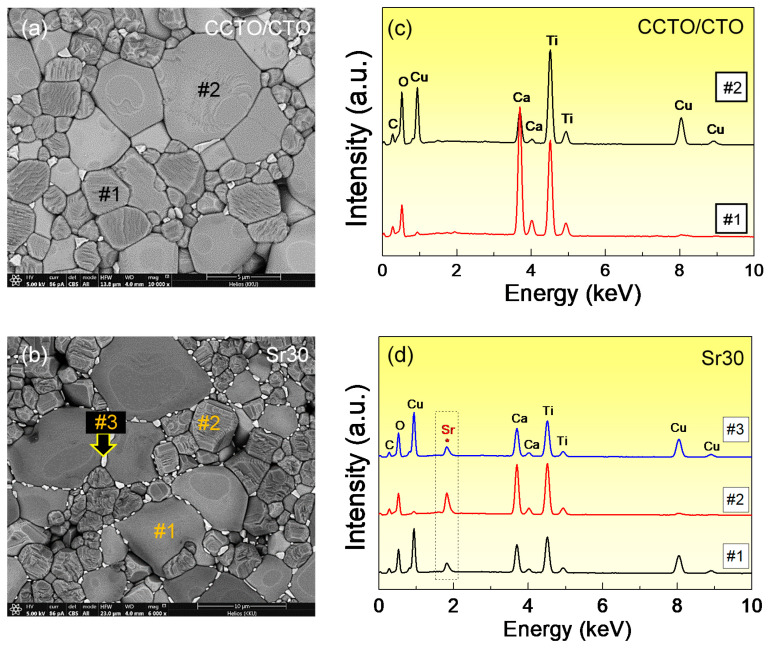
Backscattered SEM images of polished Sr^2+^-doped CCTO/CTO composite samples: (**a**) CCTO/CTO and (**b**) Sr03 composite samples. Energy-dispersive X-ray spectrometry (EDS) of (**c**) CCTO/CTO and (**d**) Sr30 composite samples detected at different points on the surface in the backscattered SEM image.

**Figure 4:**
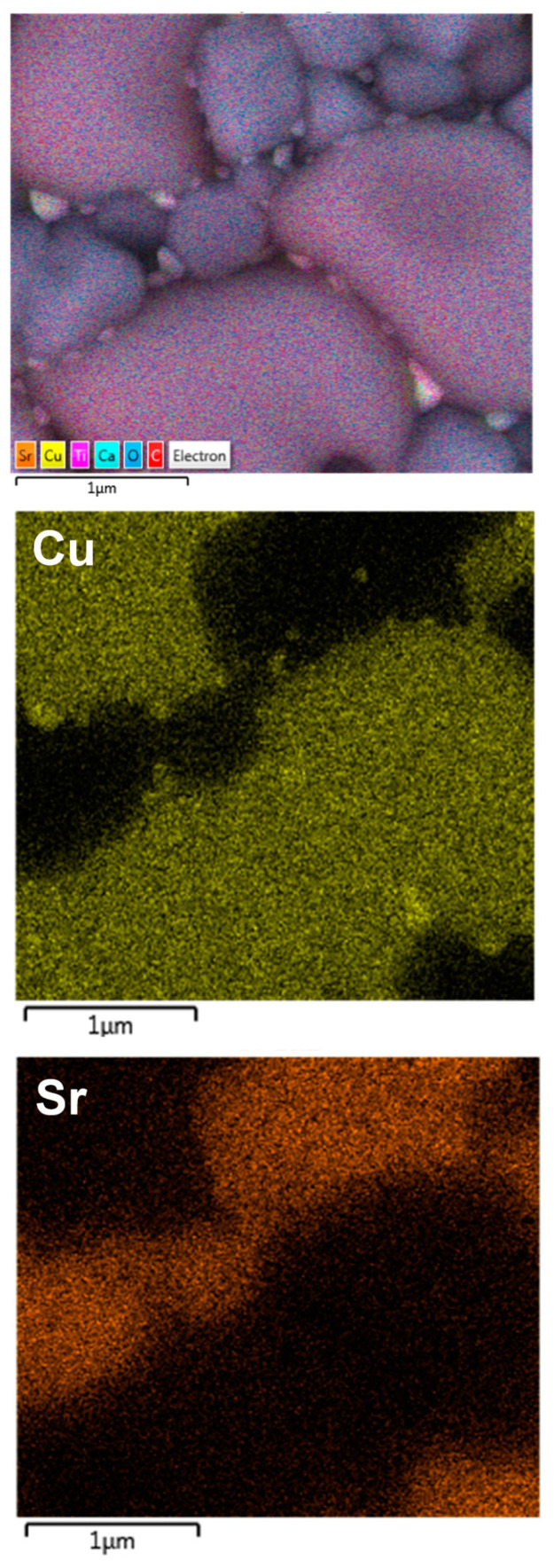
Backscattered field-emission SEM (FE-SEM) images with elemental mapping of Cu and Sr for the Sr30 composite sample.

To clearly indicate the CCTO and CTO phases in the microstructure of the ceramic composites, backscattered SEM images of the polished ceramic composites were revealed, as shown in Figure 3. As shown in Figure 3a,b, lighter grains with a smooth surface and darker grains with a rough surface were disclosed in both the CCTO/CTO and Sr^2+^-doped CCTO/CTO composites, confirming the existence of two phases as detected in the XRD patterns. Considering the atomic mass of these two phases, these phases were suggested to be the CCTO and CTO phases, respectively [18,28]. A small number of pores was observed in the CCTO/CTO composite. The number of pores tended to decrease in the Sr^2+^-doped CCTO/CTO composites. To further confirm the CCTO and CTO phases, the energy-dispersive X-ray spectroscopy (EDS) was performed in the lighter (point #2) and darker grains (point #1), as illustrated in Figure 3c,d. For the undoped CCTO/CTO composite, a Cu element was not detected in the darker grain (spectrum #1), while Ca, Ti, and O were observed, as shown in Figure 3c. Thus, the darker grain was confirmed to be the CTO phase. On the other hand, all elements of Ca, Cu, Ti, and O were detected in the lighter grain (spectrum #2), confirming the presence of the CCTO phase. To further investigate the substitution sites of Sr^2+^ doping ions, the EDS spectra of the Sr^2+^-doped CCTO/CTO ceramic composites were measured. As shown in spectrum #3 of Figure 3d, Cu-rich phase was observed as small particles (point #3). Sr^2+^ doping ions can be detected in both of the CCTO and CTO grains, as seen in spectra #1 and #2. However, the Sr^2+^ dopant was more detected in the CTO phase than that of the CCTO phase. We found that the percentages of Sr incorporated in the CTO and CCTO phases were 9.59 wt% and 0.53 wt%, respectively. As clearly confirmed by the SEM mapping images (Figure 4), an Sr element was more detected in the small grain’s region for the CTO phase, in which a Cu element could not be detected.

**Figure 5:**
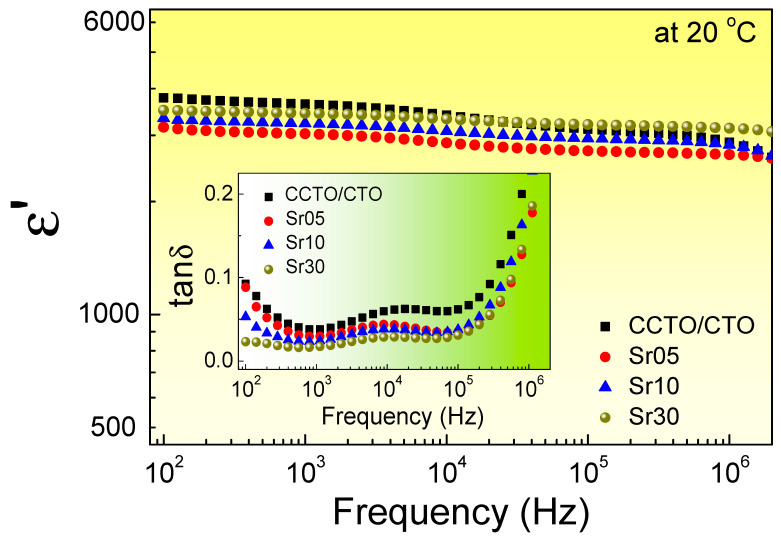
Frequency dependence of ε′ at 20 °C for all composite samples. Inset shows tanδ as a function of frequency.

Figure 5 shows the dielectric properties of the CCTO/CTO and Sr^2+^-doped CCTO/CTO ceramic composites at 20 °C. At 10^3^ Hz, the ε′ values of the CCTO/CTO, Sr05, Sr10, and Sr30 composite samples were 4247, 3511, 3742, and 3978, respectively. The ε′ of the CCTO/CTO composites slightly decreased by doping with Sr^2+^ ions, especially for the Sr30 composite sample. However, as displayed in the inset, tanδ was significantly reduced. The tanδ values at 10^3^ Hz were 0.049, 0.036, 0.028, and 0.022, respectively. The increased tanδ in the frequency range of 10^2^–10^3^ Hz was attributed to the effect of DC conduction, which resulted from a long-range motion of free charge carriers across the GBs [34,35]. On the other hand, the rapid increase in tanδ in a high-frequency range (>10^5^ Hz) was due to the dielectric relaxation process of the primary polarization, which may have been due to the polarization at the interface between the CCTO grains [36,37]. It was observed that another dielectric relaxation appeared at the middle-frequency range of ~10^4^ Hz, especially for the undoped CCTO/CTO composite. The relaxation peak of tanδ with a step-like decrease in ε′ was observed. This dielectric relaxation may have been due to the polarization relaxation at the active interface between the CCTO and CTO grains [28]. This relaxation was reduced by doping with Sr^2+^ ions.

The Sr^2+^ doping ions were better detected in the CTO grain than that in the CCTO grain. Thus, the volume fraction of the CCTO phase (f_CCTO_) in the Sr^2+^-doped CCTO/CTO composites slightly changed as the Sr^2+^ doping concentration increased. Changes in the ε′ of the Sr^2+^-doped CCTO/CTO composites were not associated with f_CCTO_, while the ε′ of Sr^2+^-doped CTO ceramics changed slightly. Variations in the ε′ of the Sr^2+^-doped CCTO/CTO composites may be attributed to the changes in the ε′ of the CCTO phase or electrically active internal interfaces. However, it was reported that the substitution of Sr^2+^ ions into the CCTO ceramics caused a decrease in ε′ [8,30]. Thus, the decreased ε′ values of the Sr05 composite sample were likely due to the significantly decreased ε′ value of the CCTO phase, which was substituted by Sr^2+^ ions. With increasing the Sr^2+^ doping concentration, the ε′ at 1 kHz slightly increased from 3511 to 3978. In this case, the effects of f_CCTO_, Sr^2+^-doped CCTO, and Sr^2+^-doped CTO phases were unlikely the origin of the observed increase in the ε′. This suggests but does not prove that slightly increased ε′ values of the Sr10 and Sr30 composite samples might have been due to the enhanced electrically active CCTO-CTO interface.

**Figure 6:**
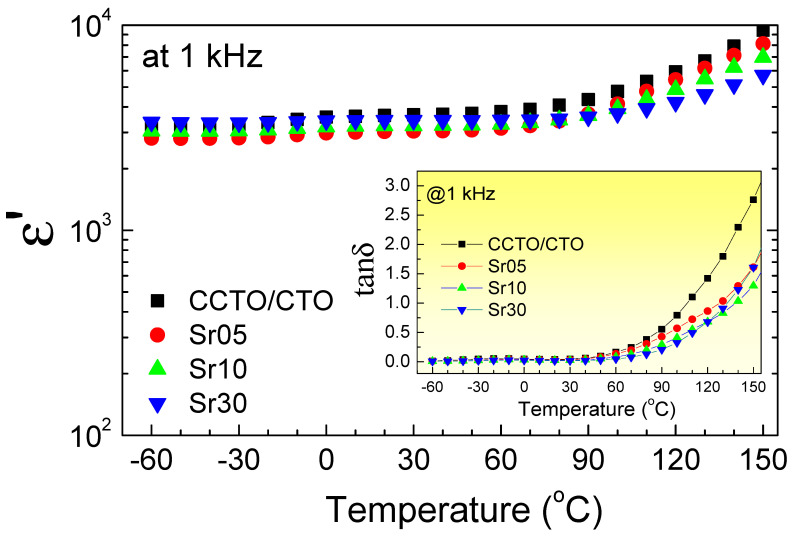
Temperature dependence of ε′ at 10^3^ Hz for all composite samples. Inset shows tanδ as a function of temperature.

The effects of Sr^2+^ doping ions on the temperature dependence of the dielectric properties of the CCTO/CTO ceramic composites are shown in Figure 6 and its inset. Notably, the Sr^2+^ doping ions can enhance the temperature stability of ε′. Furthermore, the Sr^2+^ doping ions can also suppress the increased tanδ in a high-temperature range, which is usually resulted from the increased DC conduction [35]. Thus, the substitution of the Sr^2+^ doping ions reduced the long-range motion of free charge carriers, which may be due to the increase in the GB resistance.

**Figure 7:**
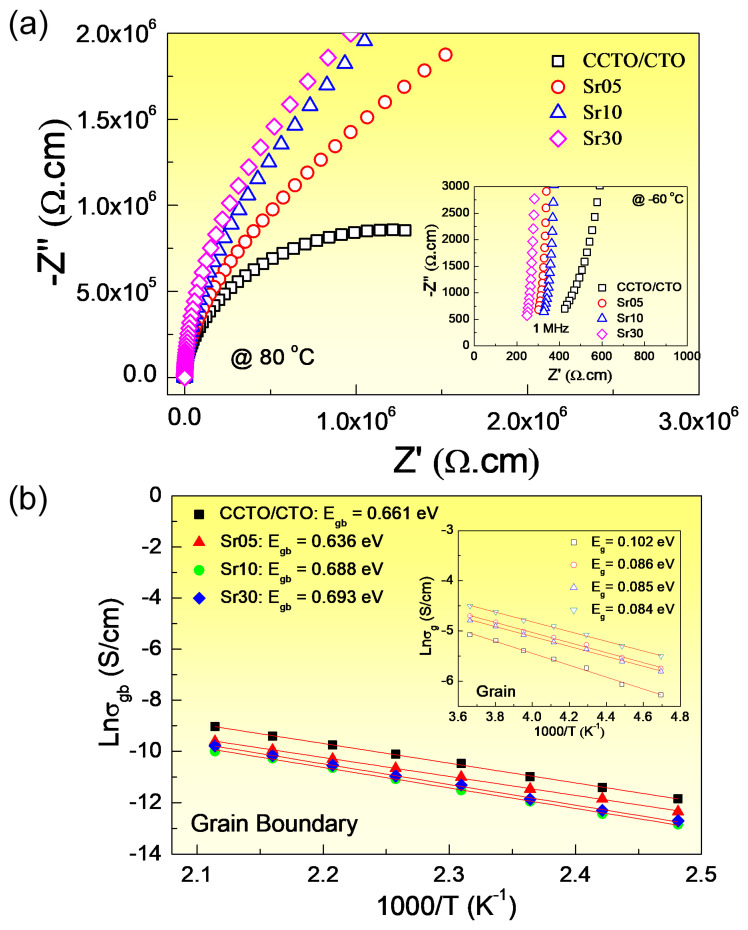
(**a**) Impedance complex plane (Z*) plots at 80 °C for all composite samples. Inset shows the nonzero intercept on the Z′ axis at −60 °C. (**b**) Arrhenius plot of the temperature dependence of grain boundaries (GB) conductivity (σ_gb_). Inset shows the Arrhenius plot of the grain conductivity (σ_g_).

To study the electrical properties of the grains and GBs in the CCTO/CTO and Sr^2+^-doped CCTO/CTO ceramic composites, impedance spectroscopy was performed. Accordingly, we can estimate the capacitance (C) and resistance (R) values of electrically active grain boundaries and grain regions. M.A. Ramirez et al. [38] demonstrated that the CCTO-CTO and CCTO-CCTO interfaces were electrically active. In contrast, the CTO-CTO interface was inactive. Thus, the results were modeled on an ideal equivalent circuit comprising two parallel RC elements connected in series. The first RC element was assigned as the electrical response of semiconducting grains in the CCTO phase. The second was assigned as the responses of the electrically active interfaces between the CCTO-CTO and CCTO-CCTO phases. Figure 7a and its inset show the impedance complex plane plots (Z*) at 80 °C and nonzero intercept at high frequencies at −60 °C, respectively. Generally, the resistance of the grain (R_g_) and GB (R_gb_) at any temperature for CCTO-based polycrystalline ceramics can be calculated from the diameters of small semicircular arc (or the nonzero intercept) and large semicircular arc in Z* plots, respectively [36]. Thus, the R_g_ and R_gb_ values at any temperature can be obtained. As clearly seen, R_gb_ of the CCTO/CTO ceramic composites was significantly increased by doping with Sr^2+^ ions, while R_g_ decreased slightly. This result is consistent with the reduction of tanδ in a low-frequency range. Furthermore, the suppressed long-range motion of free charge carriers in a high-temperature range was confirmed to originate from the significant increase in R_gb_.

The CCTO–CCTO and CCTO–CTO interfaces were found to be electrically active, giving rise to the formation of potential barriers at these interfaces [38]. A CTO–CTO interface was electrically inactive due to the insulative nature of the CTO grains [38]. Therefore, the improved electrical responses of internal interfaces in the Sr^2+^-doped CCTO/CTO ceramic composites are likely caused by the enhanced electrical responses of the CCTO–CTO and CCTO–CCTO interfaces. Variations of R_gb_ with Sr^2+^ concentrations are consistent with the observed decrease in tanδ, as illustrated in the insets of Figure 6 and Figure 7. Considering the increase in R_gb_ observed in the substituted Sr^2+^ ions, the primary factor may be due to the increase in the potential barrier height at the internal interfaces.

According to the calculated R_g_ and R_gb_ values, the conductivities of the grain (*σ*_g_) and GB (*σ*_gb_) can be calculated. As illustrated in the Figure 7b and its inset, the temperature dependence of *σ*_gb_ and *σ*_g_ follows the Arrhenius law:

(1) $$\sigma_{g/\mathrm{gb}}=\sigma_{0}\exp^{\left(\frac{-E_{g/\mathrm{gb}}}{k_{B}T}\right)}$$

where *σ*_0_ is a constant value and E_g_ and E_gb_ are the conduction activation energies inside the grains and internal interfaces (GBs), respectively. The E_g_ and E_gb_ values were calculated from the slopes. The E_gb_ values, which are associated with the potential barrier height of the GBs [39] of the CCTO/CTO, Sr05, Sr10, and Sr30 composite samples, were found to be 0.661 eV, 0.636 eV, 0.688 eV, and 0.693 eV, respectively, while E_g_ values were 0.102, 0.086, 0.085, and 0.084, respectively. The significant increase in R_gb_ value of the Sr30 composite sample was attributed to the increased potential barrier height at the GBs, which was likely due to the segregation of the Cu-rich phase at the GBs [40]. Furthermore, the substitution of Sr^2+^ doping ions may reduce the oxygen loss during the sintering process. This is one of the most important causes for the increase in the potential barrier height at the GBs [4,28,41].

**Figure 8:**
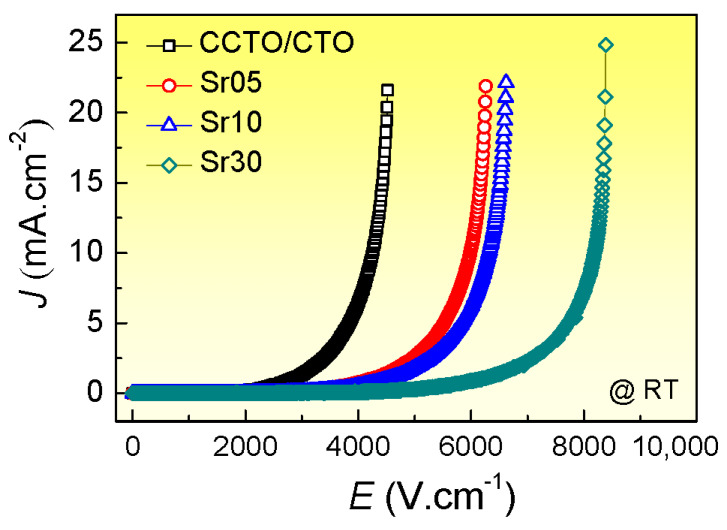
Nonlinear J-E characteristics at ~25 °C of all composite samples.

Besides the improved dielectric properties, the nonlinear J-E properties of the Sr^2+^-doped CCTO/CTO ceramic composites can also be enhanced, as shown in Figure 8. The nonlinear coefficient (α) of the CCTO/CTO, Sr05, Sr10, and Sr30 composite samples were calculated in the range of 1–10 mA/cm^2^ and found to be 5.52, 6.50, 6.86, and 7.50, respectively. Furthermore, the breakdown electric field (E_b_) values can be significantly enhanced to be 2.8 × 10^3^ V/cm, 4.2 × 10^3^ V/cm, 4.5 × 10^3^ V/cm, and 5.98 × 10^3^ V/cm, respectively. Obviously, the increased α and E_b_ values of the Sr^2+^-doped CCTO/CTO ceramic composites are consistent with an increase in R_gb_ and potential barrier height at the GBs.

## 3. Materials and Methods

### 3.1. Sample Preparation

A conventional solid-state reaction method was employed for the preparation of a Sr^2+^-doped CCTO/CTO powders with a nominal chemical composition of CCTO/CTO/xSrO powders (*x* = 0, 0.05, 0.1, and 0.3%). These ceramic compositions were referred to as the CCTO/CTO, Sr05, Sr10, Sr20, and Sr30, respectively. TiO_2_ (99.9%), CuO (99.9%), CaCO_3_ (99.9%), and SrCO_3_ (99.9%) were selected as raw materials. Initially, stoichiometric amounts of the raw materials were mixed homogeneously by ball milling in ethanol for 12 h using zirconia balls. Then, each mixed slurry was dried and then calcined in air at 900 °C for 15 h. The calcined powders were ground and pressed into disc pellets with a diameter of 9.5 mm and thickness of ~1.0 mm. To obtain the ceramic composite samples for studying properties and characterizations, the pellets were sintered at 1100 °C for 5 h.

### 3.2. Characterization

Structures and phase compositions of the sintered ceramics were studied using X–ray diffraction (XRD; PANalytical, EMPYREAN). Scanning electron microscopes (SEM; SEC, SNE-4500M) were used to reveal the microstructure and distribution of the CCTO and CTO phases. The elemental distribution of elements, i.e., Ca, Cu, Ti, O, and Sr, and backscattered electron (BSE) in the sintered ceramic were investigated using a field-emission scanning electron microscopy (FE-SEM, HITACHI SU8030) with energy-dispersive X-ray spectroscopy (EDS). Before characterization by the FE-SEM and EDS techniques, the surface of composite samples was polished and thermally etched at 1,090 °C for 1 h. The dielectric properties of the sintered ceramics were measured using a KEYSIGHT E4990A Impedance Analyzer over a frequency range of 10^2^–10^7^ Hz using an oscillation voltage of 0.5 V. The dielectric properties were measured over the range of −60–200 °C. Each step increase in measurement temperature was 10 °C with an accuracy of ±1 °C. Nonlinear J–E characteristics were determined using a high voltage measurement unit (Keithley Model 247) at RT. The E_b_ value was obtained at *J* = 1 mA·cm^−2^. The α values were calculated over the range of *J* = 1–10 mA·cm^−2^. Note that prior to electrical and dielectric measurements, Au was sputtered on each pellet face at a current of 30 mA for 8 min using a Polaron SC500 sputter coating unit (Sussex, UK).

## 4. Conclusions

The dielectric and nonlinear electrical properties of CCTO/CTO ceramic composites were successfully improved by doping with Sr^2+^ ions into the Ca^2+^ sites of the CCTO and CTO phases. Most Sr^2+^ doping ions preferred to substitute in the CTO phase rather than the CCTO phase. The CTO structure was significantly enlarged by doping with Sr^2+^ ions. The ε′ of the CCTO/CTO composites was slightly decreased from ~4.25 × 10^3^ to ~3.98 × 10^3^, while tanδ was significantly reduced from 0.05 to 0.02 due to the large decrease in σ_gb_ or increase in R_gb_. The significant increased R_gb_ value of the Sr^2+^-doped CCTO/CTO composites was caused by the increase in potential barrier height at the GBs from 0.634 eV to 0.732 eV. Significantly increased E_b_ (from 2.81 × 10^3^ to 5.98 × 10^3^ V/cm) and α (from 5.5 to 7.5) were achieved. The improved dielectric and nonlinear electrical properties were explained by the electrical responses of the active internal interfaces.

## Data Availability

The data presented in this study are available in article.

## Sample Availability

Not available.
